# Screening for Hepatitis B in partners and children of women positive for surface antigen, Burkina Faso

**DOI:** 10.2471/BLT.21.287015

**Published:** 2022-02-22

**Authors:** Alice Nanelin Guingané, Rémi Kaboré, Yusuke Shimakawa, Eric Nagaonlé Somé, Dramane Kania, Amandine Pisoni, Nicolas Nagot, Rachel King, Roger Sombié, Nicolas Meda, Philippe Van de Perre, Edouard Tuaillon

**Affiliations:** a Hepato-Gastroenterology Department, Bogodogo University Hospital Center, 01 BP 3479 Ouagadougou 01, Burkina Faso.; b Institut de Santé Publique d’Épidémiologie et du Développement, Université de Bordeaux, Bordeaux, France.; c Unité d’Épidémiologie des Maladies Émergentes, Institut Pasteur, Paris, France.; d Institut de Recherche en Sciences de la Santé, Ouagadougou, Burkina Faso.; e Centre MURAZ, Bobo Dioulasso, Burkina Faso.; f Pathogenesis and Control of Chronic and Emerging Infections, Montpellier University, Montpellier, France.; g Hepato-Gastroenterology Department, Yalgado Ouédraogo University Hospital Center, Ouagadougou, Burkina Faso.

## Abstract

**Objective:**

To evaluate the implementation of a screening strategy for the partners and children of pregnant women with hepatitis B virus (HBV) attending antenatal care.

**Methods:**

We identified pregnant women positive for HBV surface antigen (HBsAg) at antenatal consultation in Ouagadougou, Burkina Faso. At post-test counselling, women were advised to disclose their HBV status to partners and to encourage their partner and children to be screened for HBsAg. We used multivariable logistic regression to explore factors associated with uptake of screening and HBsAg positivity among family members.

**Findings:**

Of 1000 HBsAg-positive women, 436/1000 partners and 215/1281 children were screened. HBsAg was detected in 55 (12.6%) partners and 24 (11.2%) children. After adjusting for confounders, uptake of screening was higher in partners who were married, who attended the woman’s first post-test consultation and to whom the woman had disclosed her HBV status. In children, HBsAg positivity was associated with being born before the introduction of infant hepatitis B vaccination in Burkina Faso (not significant in the multivariable analysis), having a mother positive for HBV e-antigen (adjusted OR: 8.57; 95% CI: 2.49–29.48) or having a mother with HBV DNA level ≥ 200 000 IU/mL (OR: 6.83; 95% CI: 1.61–29.00).

**Conclusion:**

In low-income countries, the antenatal consultation provides a cost-effective opportunity to identify HBV-infected household contacts and link them to care. Children born before the introduction of infant hepatitis B vaccination and whose mother has higher viral load or infectivity should be a priority for testing and linkage to care.

## Résumé

**Objectif:**

Évaluer la mise en œuvre d'une stratégie de dépistage chez les partenaires et enfants de femmes enceintes porteuses du virus de l'hépatite B (VHB) faisant l'objet de soins prénatals.

**Méthodes:**

Nous avons identifié des femmes enceintes positives à l'antigène de surface du VHB (Ag HBs) dans le cadre de consultations prénatales à Ouagadougou, au Burkina Faso. Parallèlement aux conseils prodigués après le test, les femmes ont été incitées à informer leur partenaire de leur statut sérologique et à l'encourager, ainsi que leurs enfants, à se soumettre à un dépistage du Ag HBs. Nous avons employé un modèle de régression logistique multivariée pour examiner les facteurs associés à la participation au dépistage et à la positivité au Ag HBs parmi les membres d'une même famille.

**Résultats:**

Pour 1000 femmes positives au Ag HBs, 436 partenaires sur 1000 et 215 enfants sur 1281 ont été dépistés. Le Ag HBs a été détecté chez 55 partenaires (12,6%) et 24 enfants (11,2%). Après ajustement en fonction des variables confusionnelles, la participation au dépistage s'est révélée plus élevée chez les partenaires mariés, ceux ayant assisté à la première consultation post-test de la femme et ceux à qui la femme avait dévoilé son statut sérologique. Chez les enfants, la positivité au Ag HBs était liée à une naissance avant l'instauration de la vaccination des nourrissons contre l'hépatite B au Burkina Faso (non significative dans l'analyse multivariée), au fait d'avoir une mère positive à l'antigène e du VHB (OR ajusté: 8,57; IC de 95%: 2,49–29,48) ou au fait d'avoir une mère dont le taux d'ADN du VHB ≥ 200 000 IU/mL (OR: 6,83; IC de 95%: 1,61–29,00).

**Conclusion:**

Dans les pays à revenu faible, la consultation prénatale offre une occasion peu coûteuse d'identifier les contacts infectés par le VHB au sein des foyers et de favoriser leur suivi médical. Les enfants nés avant l'instauration de la vaccination des nourrissons contre l'hépatite B ainsi que ceux dont la mère présentait une plus forte charge virale ou infectiosité doivent être prioritaires en matière de dépistage et de prise en charge.

## Resumen

**Objetivo:**

Evaluar la aplicación de una estrategia de cribado para las parejas y los hijos de las mujeres embarazadas con el virus de la hepatitis** **B (VHB) que acuden a la asistencia prenatal.

**Métodos:**

Se identificaron mujeres embarazadas positivas al antígeno de superficie del VHB (HBsAg) en la consulta prenatal en Ouagadougou, Burkina** **Faso. En el asesoramiento posterior a la prueba, se recomendó a las mujeres que revelaran su estado respecto al VHB a sus parejas y que los invitaran junto con sus hijos a someterse a la prueba de detección del HBsAg. Se utilizó una regresión logística multivariable para explorar los factores asociados con la aceptación del cribado y la positividad en las pruebas del HBsAg entre los miembros de la familia.

**Resultados:**

De 1000 mujeres positivas para el HBsAg, se analizaron 436/1000 parejas y 215/1281 hijos. Se detectó el HBsAg en 55 (12,6 %) parejas y 24 (11,2 %) hijos. Luego de ajustar por variables de confusión, la aceptación del cribado fue mayor en las parejas que estaban casadas, quienes asistieron a la primera consulta de la mujer posterior a la prueba y a quienes la mujer había revelado su estado de VHB. En los niños, la positividad del HBsAg se asoció con el hecho de haber nacido antes del inicio de la vacunación infantil contra la hepatitis B en Burkina Faso (no fue significativo en el análisis multivariable), tener una madre positiva al antígeno *e* del VHB (OR ajustado: 8,57; IC del 95 %: 2,49-29,48) o tener una madre con una concentración de ADN del VHB ≥200 000 UI/ml (OR: 6,83; IC del 95 %: 1,61-29,00).

**Conclusión:**

En los países de ingresos bajos, la consulta prenatal ofrece una oportunidad rentable para identificar a los contactos familiares infectados por el VHB y vincularlos a la atención. Los niños nacidos antes del inicio de la vacunación infantil contra la hepatitis B y cuyas madres tienen una carga viral o infectividad más elevada deberían ser objeto prioritario de pruebas de detección y vinculación a la atención sanitaria.

## ملخص

الغرض تقييم تنفيذ استراتيجية فحص للشركاء وأطفال النساء الحوامل المصابات بفيروس الالتهاب الكبدي ب (HBV) الذين يحضرون الرعاية السابقة للولادة.

الطريقة قمنا بتحديد النساء الحوامل المصابات بالمستضد السطحي للالتهاب الكبدي ب (HBsAg) في استشارة ما قبل الولادة في واغادوغو، بوركينا فاصو. في استشارة ما بعد الفحص، تم نصح النساء بالكشف عن حالة الإصابة بالالتهاب الكبدي ب لديهن لشركائهن، وتشجيع شركائهن وأطفالهن على الخضوع لفحص المستضد السطحي للالتهاب الكبدي ب. استخدمنا التحوف اللوجيستي متعدد المتغيرات لاستكشاف العوامل المرتبطة بالخضوع للفحص، والمستضد السطحي للالتهاب الكبدي ب بين أفراد الأسرة.

النتائج من بين 1000 امرأة مصابة بالمستضد السطحي للالتهاب الكبدي ب، تم فحص 436/1000 شريك، و215/1281 طفلاً. تم اكتشاف المستضد السطحي للالتهاب الكبدي ب في 55 شريكًا (12.6%)، و24 طفلاً (11.2%). بعد التكيف مع المتغيرات المحيّرة، كان معدل الخضوع للفحص أعلى لدى الشركاء المتزوجين، والذين حضروا الاستشارة بعد أول فحص للمرأة، والذين كشفت لها المرأة عن حالة إصابتها بالتهاب الكبد ب. أما في الأطفال، فقد ارتبطت الإصابة بالمستضد السطحي للالتهاب الكبدي ب بالولادة قبل طرح لقاح التهاب الكبد ب للرضع في بوركينا فاصو (ليس مهمًا في التحليل متعدد المتغيرات)، مع وجود أم مصابة بمستضد التهاب الكبد ب الإلكتروني (نسبة الاحتمالات المعدلة: 8.57؛ ‏بفاصل ثقة 95%: 2.49 إلى 29.48)، أو وجود أم مصابة بالتهاب الكبد ب المستوى DNA ≥200000 وحدة دولية/مل (نسبة الاحتمالات: 6.83؛ ‏بفاصل ثقة 95%: 1.61 إلى 29.00).

الاستنتاج في الدول ذات الدخل المنخفض، توفر استشارة ما قبل الولادة فرصة فعالة من حيث التكلفة لتحديد جهات الاتصال المنزلي المصابة بفيروس التهاب الكبد ب، وربطها بالرعاية. إن الأطفال المولودين قبل طرح لقاح التهاب الكبد ب للرضع، والذين كانت أمهاتهم تحملن حمولة فيروسية أعلى أو إصابة أقوى بالعدوى، يجب أن تكون لهم أولوية للاختبار والربط بالرعاية..

## 摘要

**目的:**

评估对参加产前检查的孕妇的伴侣和子女进行乙肝病毒 (HBV) 筛查的实施情况。

**方法:**

我们在布基纳法索瓦加杜古的产前咨询中发现了 HBV 表面抗原 (HBsAg) 呈阳性的孕妇。在检测后的咨询中，我们建议这些妇女向其伴侣告知其 HBV 状况，并鼓励其伴侣和子女进行 HBsAg 筛查。我们使用多变量逻辑回归方法来探索家庭成员接受筛查且 HBsAg 呈阳性相关的因素。

**结果:**

在 1000 名 HBsAg 呈阳性的女性中，436/1000 名伴侣和 215/1281 名儿童接受了筛查。有 55 名（12.6%）伴侣和 24 名（11.2%）儿童检测为 HBsAg 呈阳性。在对混杂因素进行调整后，已婚妇女的伴侣、检测后参加首次询诊的妇女的伴侣以及向其伴侣告知 HBV 状况的妇女的伴侣进行筛查的比率更高。在儿童中，布基纳法索地区 HBsAg 呈阳性与婴儿在进行乙肝疫苗接种前出生（在多变量分析中不显著），母亲 HBV e 抗原呈阳性（调整后的 OR：8.57；95% 置信区间：2.49–29.48），或者母亲 HBV DNA 水平为³200,000 IU/毫升（OR：6.83；95% 置信区间：1.61–29.00）有关。

**结论:**

在低收入国家，产前咨询提供了经济有效的机会以确认感染 HBV 的家庭接触者并使其接受护理。对于母亲的病毒载量或传染性较高且在接受乙肝疫苗接种前出生的婴儿，应优先进行检测并使其接受护理。

## Резюме

**Цель:**

Оценить осуществление стратегии скрининга для партнеров и детей беременных женщин, положительных по вирусу гепатита В (HBV), которые посещают учреждения дородовой помощи.

**Методы:**

Авторы выявляли беременных женщин, положительных по поверхностному антигену HBV (HBsAg), в женской консультации г. Уагадугу, Буркина-Фасо. На консультации после тестирования женщинам было рекомендовано сообщить о своем HBV-статусе партнеру и привести партнера и детей на скрининг по гепатиту В. Использовалась многопеременная логистическая регрессия для изучения факторов, ассоциируемых с тем, как члены семьи были охвачены скринингом и насколько часто выявлялась положительность по HBsAg.

**Результаты:**

Для 1000 положительных по HBsAg женщин скрининг прошли 436/1000 партнеров и 215/1281 ребенок. HBsAg обнаружили у 55 партнеров (12,6%) и у 24 детей (11,2%). После внесения поправки на факторы, затрудняющие оценку причинно-следственной связи, оказалось, что охват скринингом был выше у женатых мужчин, которые пришли с супругами на первую консультацию после тестирования и которым жены рассказали о своем положительном статусе. У детей положительная реакция в тесте на HBsAg была связана с фактом рождения до начала кампании вакцинирования младенцев от гепатита В в Буркина-Фасо (незначимо для многопеременного анализа), с положительностью матери по е-антигену HBV (скорректированное ОШ: 8,57; 95%-й ДИ: 2,49–29,48) или с вирусной нагрузкой у матери по HBV ≥ 200 000 МЕ/мл (ОШ: 6,83; 95%-й ДИ: 1,61–29,00).

**Вывод:**

В странах с низким уровнем дохода женская дородовая консультация позволяет экономичным образом выявить бытовые контакты инфицированных HBV лиц и дать им возможность получения медицинской помощи. Дети, родившиеся до начала кампании вакцинирования младенцев от гепатита В, и те, у кого матери имели более высокую вирусную нагрузку, должны проходить тестирование и приступать к лечению в первоочередном порядке.

## Introduction

In 2016, an estimated 257 million people worldwide were chronically infected with hepatitis B virus (HBV), of whom only 27 million (10.5%) were aware of their infection.^1^ Chronic HBV infection is highly endemic in sub-Saharan Africa, where it is transmitted from mother to child at birth or horizontal transmission among children and family members.^2^^–^^4^ In 2016, the World Health Organization (WHO) Member States, including Burkina Faso, approved three global health sector strategies to guide action against human immunodeficiency virus, viral hepatitis and sexually transmitted infections.^5^ Eliminating HBV as a global public health problem by 2030 is one of the key goals of the WHO agenda.^6^ The main measures to achieve this objective in Africa include the prevention of mother-to-child transmission through the universal implementation of the hepatitis B birth dose vaccine and antiviral treatment of HBV-infected mothers who have high viral loads during the third trimester of pregnancy.^7^^,^^8^ A key aim is to create a new African generation free of HBV through the prevention of HBV mother-to-child transmission. It is also important to identify people who are chronically infected with HBV and to treat those with an increased risk to prevent life-threatening complications such as cirrhosis, liver failure and hepatocellular carcinoma.^9^ WHO recommends focused testing of high-risk groups, such as children and close household contacts of HBV-infected people, followed by linking them to care and treatment services.^2^ Antenatal consultation, therefore, may provide a unique opportunity to identify additional cases of HBV infection in family members of infected pregnant women.

Burkina Faso is a low-income country where hepatitis B is highly endemic. Vaccination against hepatitis B was introduced in 2006 in the expanded programme of immunization. According to the United Nations Children’s Fund and WHO, the coverage of three doses of hepatitis B vaccination, scheduled at 8, 12 and 16 weeks of life, has been consistently over 90% since 2009.^10^ Children born to HBV-infected mothers often become chronic carriers of HBV surface antigen (HBsAg).^11^^–^^13^ In West Africa, hepatitis B is a main contributor to cirrhosis and liver cancer, with about 2 million disability-adjusted life-years attributable to viral hepatitis.^14^ However, a low proportion of HBV-infected people, estimated to be 0.3% in 2015, has been diagnosed in Africa.^15^ Although it has not yet been integrated into the expanded programme of immunization, hepatitis B birth dose vaccine will be introduced during 2022. In July 2017, a national strategic plan to control viral hepatitis was adopted in the country.

In this implementation research in Ouagadougou, Burkina Faso, we assessed the feasibility of a screening programme at antenatal care facilities targeting the partners and children of pregnant women identified as carriers of HBsAg. We explored the sociodemographic characteristics associated with the successful uptake of screening by partners and children and the factors associated with the risk of HBV infection in children.

## Methods

### Setting

In 2014 a programme for the prevention of mother-to-child transmission of HBV was introduced in the Baskuy district of Ouagadougou.^16^ The district comprises nine primary-care centres serving an estimated 287 000 people and one tertiary referral hospital: the Yalgado Ouédraogo University Hospital Center. As part of the programme, women attending antenatal care in any of the primary-care centres in Baskuy district are systematically offered screening for HBsAg. 

### Intervention

The screening programme included the following four steps: (i) training on HBV counselling for health-care workers in primary-care services; (ii) counselling and offer of HBsAg screening for pregnant women during the first antenatal consultation in the primary-care centre; (iii) simplified referral process of women testing positive for HBsAg to the hepato-gastroenterology department of the referral hospital; and (iv) post-test counselling of HBsAg-positive women at the referral hospital. 

Post-test counselling with a hepatologist and a study nurse took approximately 25 minutes for each woman and included an explanation of the disease and the potential risk of transmission to her baby and the rest of her family. Women were advised to undertake hepatitis B e-antigen (HBeAg) and HBV deoxyribonucleic acid (DNA) testing to assess their eligibility for antiviral therapy. Women were also advised to disclose their infection status to their partner and to invite their children and partner for HBV screening. 

A total of six visits at the referral hospital were planned for HBsAg-positive women: three during pregnancy (the first visit, and 1 and 2 months after the first visit), and another three visits after delivery (week 2, month 3 and month 6 postpartum). At each follow-up visit, a study physician reminded the women about the screening of family members. The vaccination status of the women’s children was ascertained using the vaccination record or by interviewing the mothers. As part of the routine care, women were informed of the benefit of HBV vaccination for unvaccinated children who were born before the introduction of HBV vaccination in 2006. Partners testing positive for HBsAg were informed of the potential benefit of treatment of chronic HBV infection if they were eligible for antiviral therapy. Partners testing negative for HBsAg were informed of the benefit of vaccination if they were negative for hepatitis B core antibody. 

### Implementation study

We recruited a total of 1000 HBsAg-positive women to the study cohort (index cases). We calculated the sample size assuming that 50% of their partners would accept and undertake HBV screening; this sample size would therefore give us a precision of ± 3% for our primary outcome of the HBV screening uptake rate in partners. The study was approved by the national ethics committee (reference number 2017–11–164).

The study started in September 2014 and ended in September 2019, 6 months after we completed the enrolment of 1000 women. Women arrived at different stages in their pregnancy and each woman was followed up to 6 months of infant life. We analysed the data from September 2019 to May 2021. The current analysis included all HBsAg-positive pregnant women evaluated at the referral hospital, irrespective of whether they could complete the biological tests recommended by the study staff. 

Data for the study were collected by research assistants during the post-test counselling interviews with mothers. We analysed sociodemographic and clinical data from the woman and her partner for the following variables: age, education level, marital status and occupation. We also included the following variables for women: number of previous pregnancies; gestational age at baseline; HBeAg serological result; HBV DNA level; retention in care; whether they could complete the payment for the examinations related to pregnancy and HBV management; awareness of HBV status before the index screening; disclosure of HBV status to partner after the screening; and attendance at post-test screening as a couple.

Biological analyses for women, partners and children were carried out at the Cerba laboratory in Ouagadougou. HBsAg status (positive or negative) was determined using an enzyme-linked fluorescent assay (VIDAS®, bioMérieux, Marcy-l’Étoile, France). HBeAg status (positive or negative) was determined using a rapid diagnostic test (SD Bioline, Standard Diagnostics, Suwon, Republic of Korea). For the quantification of HBV DNA (IU/mL) we used the Cobas®TaqMan® HBV test (Roche, Basel, Switzerland). Study participants paid the cost of laboratory tests: 3.88 United States dollars (US$) for the HBsAg test, US$ 3.88 for the HBeAg test and US$ 37.02 for HBV DNA quantification.

### Statistical analysis

The primary outcomes of the study were the uptake of HBV screening and the seroprevalence of HBsAg among partners and children of HBsAg-positive women. The secondary outcomes were the sociodemographic and biological factors associated with HBV infection in partners and children. We used univariable and multivariable logistic regression analyses to identify factors associated with the successful uptake of screening and factors associated with HBsAg positivity in the women’s partner and children. All the variables significantly associated (*P* < 0.05) in the univariable analysis were further assessed in the multivariable model. Using a backward stepwise regression, we selected the final multivariable model. We made a complete case analysis by excluding those with missing data. All the analyses were performed using R version 3.4.2 in R studio (R Foundation for Statistical Computing, Vienna, Austria).

## Results

### Characteristics of index women

We recruited a total of 1000 HBsAg-positive women (index cases) to the study (Table 1). The prevalence of positive HBeAg in the study group was 10.3% (71/689 women) and the prevalence of high viral load (HBV DNA ≥ 200 000 IU/mL) was 9.5% (59/623 women). 

**Table 1:** Characteristics of HBsAg-positive pregnant women recruited to the study of family screening for hepatitis B virus in Baskuy district, Ouagadougou, Burkina Faso, 2014–2019

Characteristic	No. (%)
**Woman’s age, years (*n* = 1000)**	
16–22	235 (23.5)
23–29	404 (40.4)
30–36	284 (28.4)
37–43	77 (7.7)
**Woman’s level of education (*n* = 974)**	
No education	244 (25.0)
Primary	188 (19.3)
Secondary	401 (41.1)
Superior	141 (14.4)
**Woman’s occupation (*n* = 999)**	
Housewife/Farmer	483 (48.3)
Student	148 (14.8)
Informal sector	180 (18.0)
Saleswoman	120 (12.0)
Civil servant	68 (6.8)
**Marital status (*n* = 1000)**	
Married	960 (96.0)
Single	40 (4.0)
**No. of children (*n* = 1000)**	
0	375 (37.5)
1	238 (23.8)
2	214 (21.4)
3	111 (11.1)
4	35 (3.5)
5	21 (2.1)
6	5 (0.5)
7	1 (0.1)
**No. of pregnancies (*n* = 1000)**	
1	375 (37.5)
2–4	563 (56.3)
> 4	62 (6.2)
**Baby’s gestational age (*n* = 954)**	
First trimester	122 (12.8)
Second trimester	484 (50.7)
Third trimester	348 (36.5)
**Timely HBV vaccine birth dose < 24 hours in neonate (*n* = 592)**	
Yes	521 (88)
No	71 (12)
**Disclosed HBV-positive status to partner (*n* = 1000)**	
Yes	886 (88.6)
No	114 (11.4)
**Attended first post-test specialist consultation with partner (*n* = 1000)**	
Yes	497 (49.7)
No	503 (50.3)
**Knew about HBV-positive status before first screening (*n* = 1000)**	
Yes	30 (3.0)
No	970 (97.0)
**HBeAg status (*n* = 689)**	
Negative	618 (89.7)
Positive	71 (10.3)
**HBV DNA level, IU/mL (*n* = 623)**	
< 15	155 (24.9)
15–1999	305 (48.9)
2000–199 999	104 (16.7)
≥ 200 000	59 (9.5)
**Transaminases level, IU/mL (*n* = 701)**	
0–40	681 (97.1)
40–80	10 (1.4)
80–160	4 (0.6)
160– 240	2 (0.3)
240–1700	4 (0.6)

HBeAg: hepatitis B e-antigen; HBsAg: hepatitis B surface antigen; HBV: hepatitis B virus; HBV DNA: hepatitis B virus deoxyribonucleic acid assay.

Note: Inconsistencies arise in some values due to rounding.

**Fig. 1:**
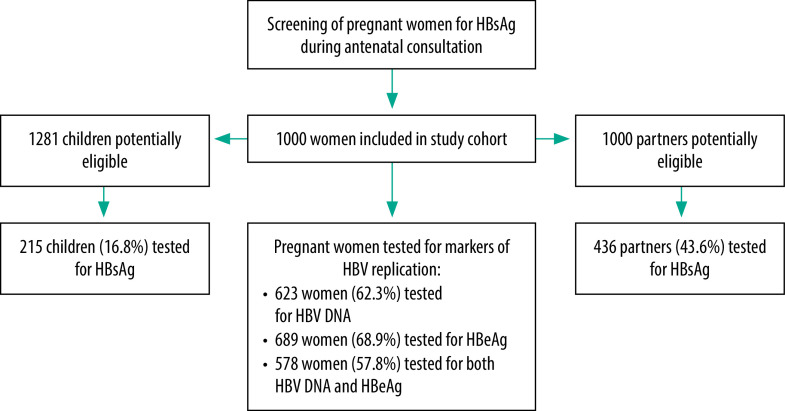
Flowchart of the study of family screening for hepatitis B, Baskuy district, Ouagadougou, Burkina Faso, 2014–2019

HBeAg: hepatitis B e-antigen; HbsAg: hepatitis B surface antigen; HBV: hepatitis B virus; HBV DNA: hepatitis B virus deoxyribonucleic acid assay.

**Fig. 2:**
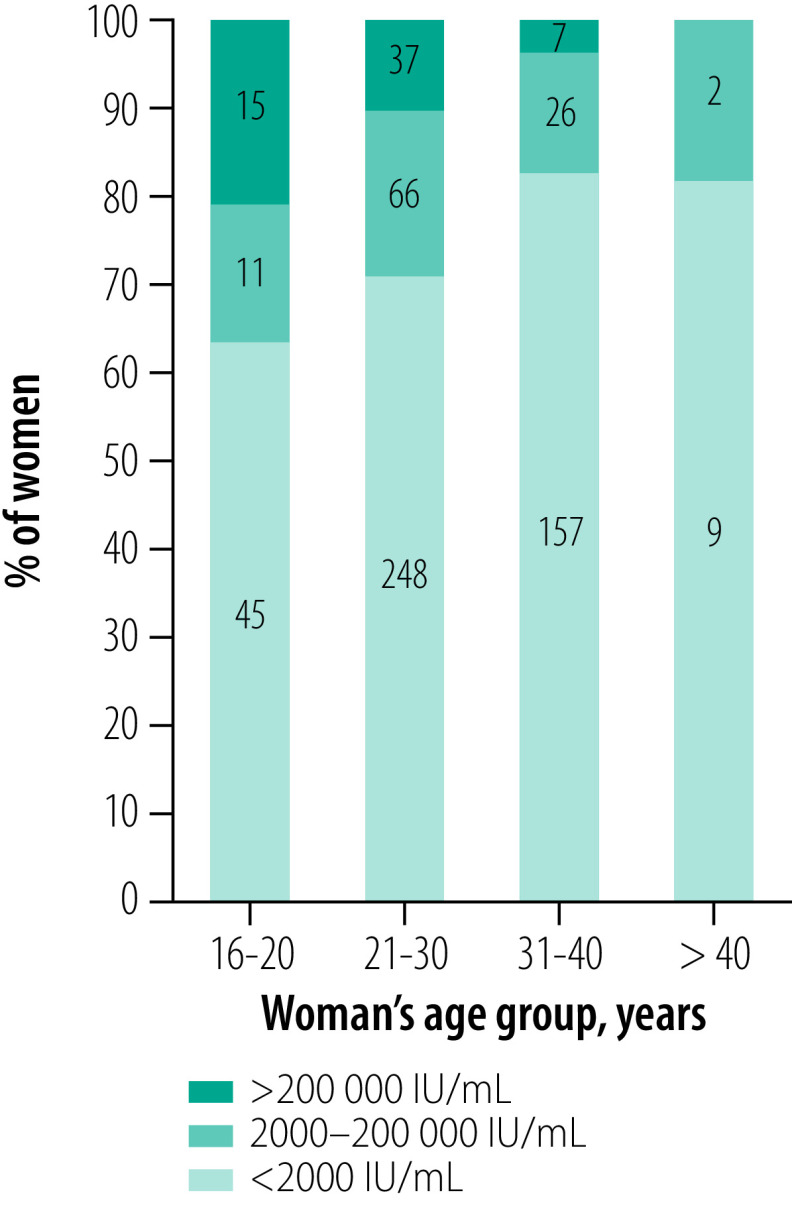
HBV DNA levels in pregnant women with hepatitis B virus infection by age group, Burkina Faso, 2014–2019

**Fig. 3:**
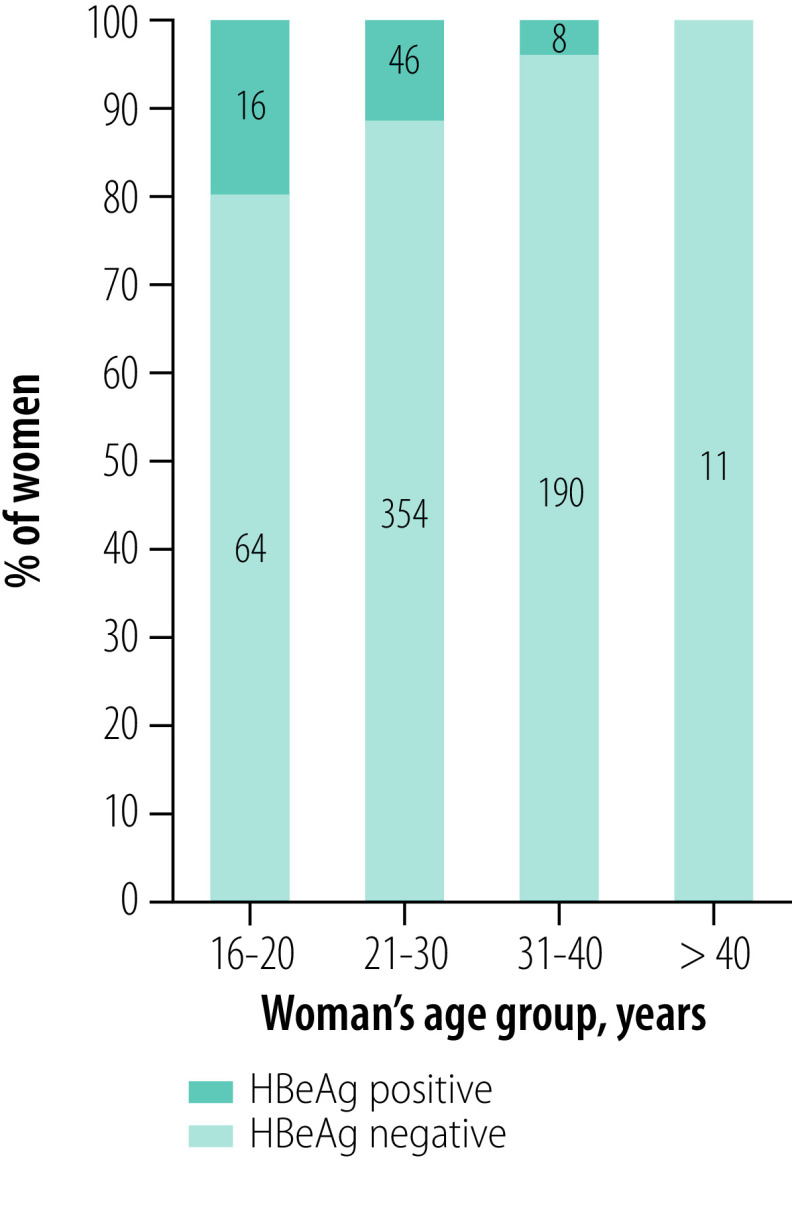
HBeAg-positivity in pregnant women with hepatitis B virus infection by age group, Burkina Faso, 2014–2019

**Fig. 4:**
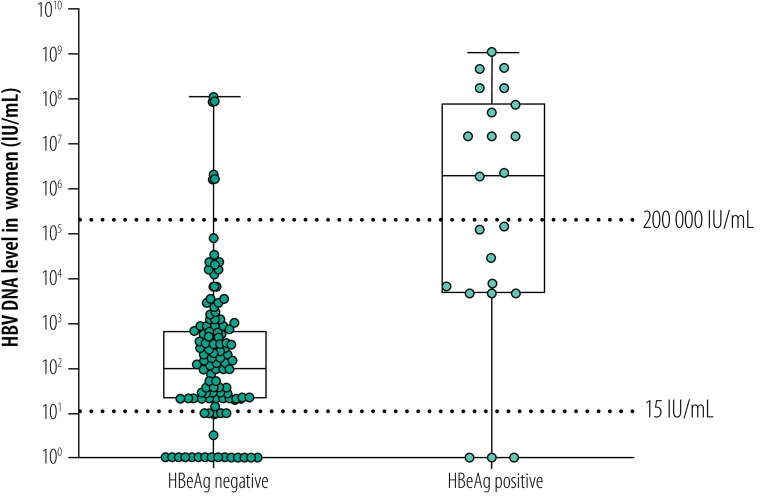
Distribution of HBV DNA levels and HBeAg positivity in pregnant women, Burkina Faso, 2014–2019

HBeAg: hepatitis B e-antigen; HBV DNA: hepatitis B virus deoxyribonucleic acid assay; IU: international unit.

Note: A total of 516 women were tested for both HBeAg and HBV DNA. The boxplot shows the median values and the first and third quartiles; the error bars show the first and ninth deciles. Lower dotted line indicates the lower limit of detection of HBV DNA; upper dotted line indicates the threshold of 200 000 IU/mL, below which the risk of failure of HBV mother-to-child immunoprophylaxis is negligible.^17^

A total of 578 women (57.8%) agreed to have both HBV DNA quantification and HBeAg testing (Fig. 1). Among 62 HBeAg-positive women, 38 women (61.3%) had HBV DNA level ≥ 200 000 IU/mL. In 516 HBeAg-negative women, a small proportion of women (3.5%; 18 women) had HBV DNA level ≥ 200 000 IU/mL. The prevalence of high HBV DNA levels ≥ 200 000 IU/mL (Fig. 2) and of HBeAg-positivity (Fig. 3) gradually decreased with increasing age of women (*P* < 0.001 and *P* = 0.01, respectively; Fisher exact test). Women who were carriers of HBeAg and with HBV DNA level ≥ 200 000 IU/mL were mainly younger than 30 years; the prevalence of HBeAg was particularly high in women younger than 20 years (20.0%; 16/80 women; Fig. 3). HBeAg-positive women had higher HBV DNA levels than HBeAg-negative women (*P* < 0.001, Fig. 4). 

HBV DNA: hepatitis B virus deoxyribonucleic acid assay; IU: international unit.

Note: Data values in columns are the numbers of women.

HBeAg: hepatitis B e-antigen.

Note: Data values in columns are the numbers of women. *P* < 0.001 (Fisher exact test) for relationship between age and HBeAg status.

### Uptake of screening by family members

A total of 2281 eligible family members were identified from the index women, including 1000 partners and 1281 children. Most of the HBV-infected women (88.6%; 886 women) had disclosed their infection status to their partners. A total of 651 family members were screened, including 436 of the partners (43.6%) and 215 of the children (16.8%). The distribution of partners and children screening HBsAg positive by household size (the number of children) is shown in Table 2.

**Table 2:** Screening of family members of pregnant women with hepatitis B virus infection, by household size in Baskuy district, Ouagadougou, Burkina Faso, 2014–2019

No. of children per household	No. of index women (*n* = 1000)	Partners of index women (*n* = 1 000)			Children of index women (*n* = 1 281)			Children and partners of index women (*n* = 2 281)	
		No. screened	No. (%) HBsAg positive		No. screened	No. (%) HBsAg positive		No. screened	No. (%) HBsAg positive
0	375^a^	174	24 (13.8)		NA	NA		174	24 (13.8)
1	238	106	15 (14.1)		56	6 (10.7)		162	21 (12.9)
2	214	93	10 (10.7)		77	9 (11.7)		170	19 (11.2)
3	111	44	4 (9.1)		60	3 (5.0)		104	7 (6.7)
4	35	12	2 (16.6)		15	4 (26.7)		27	6 (22.2)
5	21	6	0 (0.0)		7	2 (28.6)		13	2 (15.4)
6	5	1	0 (0.0)		0	0 (0.0)		1	0 (0.0)
7	1	0	0 (0.0)		0	0 (0.0)		0	0 (0.0)
**Total**	**NA**	**436**	**55 (12.6)**		**215**	**24 (11.2)**		**651**	**79 (12.1)**

HBsAg: hepatitis B surface antigen; NA: not applicable.

^a^ Women currently pregnant with first child.

The factors associated with the uptake of screening are presented in Table 3 for the partners and Table 4 for children. After adjusting for confounding factors in the multivariable analysis, uptake of screening by partners was significantly higher in married couples; in couples with a higher level of education; when the woman was retained in antenatal care (attended five or more consultations); in partners who attended the first post-test specialist consultation; and in partners to whom the women had disclosed her HBV status. Maternal factors significantly associated with higher uptake of screening for children were: higher education (adjusted OR: 2.91; 95% confidence interval, CI: 1.42–5.94); greater number of pregnancies (adjusted OR for > 4 pregnancies: 13.78; 95% CI: 5.40–35.13); retention in care (adjusted OR: 3.27; 95% CI: 2.14–4.98) and sharing of HBV status with her partner (adjusted OR: 2.81; 95% CI: 1.16–6.80).

**Table 3:** Factors associated with uptake of screening by the partners of pregnant women with hepatitis B virus infection in Baskuy district, Ouagadougou, Burkina Faso, 2014–2019

Variable	Total no. of index women (*n* = 1000)	No. (%) of women whose partner was screened	Crude OR (95% CI)	Adjusted OR (95% CI)
**Woman’s age, years**				
≤ 22	235	100 (42.6)	1.00	NA
23–29	404	184 (45.5)	1.13 (0.81–1.56)	NA
30–36	284	117 (41.2)	0.93 (0.66–1.33)	NA
≥ 37	77	35 (45.4)	1.09 (0.65–1.83)	NA
**Woman’s level of education**				
No education	244	84 (34.4)	1.00	NA
Primary	188	63 (33.5)	0.96 (0.64–1.44)	0.97 (0.61–1.54)
Secondary	401	188 (46.9)	1.93 (1.38–2.69)	1.75 (1.16–2.65)
Higher	141	82 (58.2)	2.65 (1.73–4.05)	2.19 (1.21–3.96)
**Woman’s occupation**				
Housewife or farmer	483	194 (40.2)	1.00	NA
Saleswoman	120	50 (41.7)	1.05 (0.70–1.57)	NA
Student	148	76 (51.4)	1.57 (1.09–2.28)	NA
Civil servant	68	41 (60.3)	2.26 (1.35–3.80)	NA
Informal sector	180	75 (41.7)	1.06 (0.75–1.51)	NA
**No. of pregnancies**				
1	375	147 (39.2)	1.00	NA
2–4	563	237 (42.1)	0.87 (0.66–1.15)	NA
> 4	62	35 (56.5)	0.57 (0.36–0.91)	NA
**Baby’s gestational age**				
First trimester	122	69 (56.5)	1.00	NA
Second trimester	484	229 (47.3)	0.69 (0.46–1.03)	NA
Third trimester	348	121 (34.8)	0.41 (0.27–0.62)	NA
**Marital status**				
Married	960	427 (44.5)	1.00	NA
Not married	40	8 (20.0)	0.33 (0.15–0.73)	0.21 (0.09–0.53)
**Partner’s age, years**				
≤ 22	13	5 (38.5)	1.00	NA
23–29	208	89 (42.8)	1.20 (0.38–3.78)	NA
30–36	402	178 (44.3)	1.27 (0.41–3.94)	NA
≥ 37	237	164 (69.2)	1.35 (0.43–4.22)	NA
**Partner’s level of education**				
No education	309	109 (35.3)	1.00	NA
Primary	255	104 (40.8)	1.27 (0.90–1.79)	1.09 (0.73–1.64)
Secondary	357	167 (46.8)	1.62 (1.19–2.22)	1.18 (0.79–1.76)
Higher	76	56 (73.7)	5.16 (2.95–9.05)	3.62 (1.73–7.58)
**Partner’s occupation**				
Informal sector or subordinate manager	688	270 (39.2)	1.00	NA
Middle or senior manager	265	152 (57.4)	2.19 (1.57–2.78)	NA
Student	28	11 (39.3)	1.00 (0.46–2.18)	NA
Unemployed	14	3 (21.4)	0.42 (0.12–1.53)	NA
**Retention of woman in care**				
Attended < 5 visits	708	227 (32.1)	1.00	NA
Attended ≥ 5 visits	292	209 (71.6)	5.90 (4.48–7.76)	4.84 (3.50–6.69)
**Family able to cover expenses of tests**				
No	74	32 (43.2)	1.00	NA
Yes	338	218 (64.5)	2.38 (1.43–3.98)	2.04 (1.15–3.61)
Missing data	588	186 (31.6)	0.61 (0.37–0.99)	0.93 (0.53–1.62)
**Attended first post-test specialist consultation with partner**				
No	503	195 (38.8)	1.00	NA
Yes	497	241 (48.5)	1.49 (1.16–1.92)	1.61 (1.18–2.20)
**Knew about HBV status before first screening**				
No	918	391 (42.6)	1.00	NA
Yes	82	44 (53.7)	2.30 (1.08–4.87)	NA
**Disclosed HBV-positive status to partner**				
No	114	25 (21.9)	1.00	NA
Yes	886	411 (46.4)	3.08 (1.94–4.89)	2.86 (1.68–4.88)

CI: confidence interval; HBV: hepatitis B virus; NA: not applicable; OR: odds ratio.

Notes: We included the following variables in the multivariable analysis: baby’s gestational age, partner’s age, woman’s age, knew about HBV-positive status before first screening, number of pregnancies, woman’s occupation, partner’s occupation, retention of woman in care, attended first post-test specialist consultation with partner, disclosed HBV-positive status to partner. In some instances the values do not add up to the sample size due to missing data.

**Table 4:** Factors associated with uptake of screening by children of pregnant women with hepatitis B virus infection in Baskuy district, Ouagadougou, Burkina Faso, 2014–2019

Variable	Total no. of index women (*n* = 1000)	No. (%) of women whose children were screened	Crude OR (95% CI)	Adjusted OR (95% CI)
**Woman’s age, years**				
≤ 22	235	14 (5.9)	1.00	NA
23–29	404	48 (11.9)	2.12 (1.14–3.94)	NA
30–36	284	51 (17.9)	3.51 (1.89–6.50)	NA
≥ 37	77	22 (28.6)	6.17 (2.97–12.82)	NA
**Woman’s level of education**				
No education	244	21 (8.6)	1.00	1.00
Primary	188	26 (13.8)	1.63 (0.88–3.01)	1.83 (0.94–3.51)
Secondary	401	65 (16.2)	2.13 (1.26–3.57)	2.83 (1.61–4.95)
Higher	141	21 (14.9)	1.86 (0.97–3.53)	2.91 (1.42–5.94)
**Woman’s occupation**				
Housewife or farmer	483	52 (10.8)	1.00	NA
Saleswoman	120	16 (13.3)	1.24 (0.68–2.24)	NA
Student	148	17 (11.5)	1.05 (0.58–1.88)	NA
Civil servant	68	17 (25.0)	2.70 (1.45–5.02)	NA
Informal sector	180	33 (18.3)	1.82 (1.14–2.92)	NA
**No. of pregnancies**				
1	375	7 (1.9)	1.00	1.00
2–4	563	102 (18.1)	10.11 (4.63–22.04)	12.32 (5.57–27.25)
> 4	62	20 (32.3)	10.49 (4.28–25.65)	13.78 (5.40–35.13)
**Baby’s gestational age**				
First trimester	122	15 (12.3)	1.00	NA
Second trimester	484	69 (14.2)	1.20 (0.66–2.18)	NA
Third trimester	348	47 (13.5)	1.11 (0.59–2.07)	NA
**Marital status**				
Not married	40	1 (2.5)	1.00	NA
Married	960	132 (13.7)	0.19 (0.02–1.23)	NA
**Partner’s age, years**				
≤ 22	13	1 (7.7)	1.00	NA
23–29	208	21 (10.1)	1.35 (0.16–10.9)	NA
30–36	402	47 (11.7)	1.58 (0.20–12.5)	NA
≥ 37	237	66 (27.8)	2.78 (0.35–21.6)	NA
**Partner’s level of education**				
No education	309	30 (9.7)	1.00	NA
Primary	255	34 (13.3)	1.49 (0.88–2.49)	NA
Secondary	357	59 (16.5)	1.85 (1.15–2.95)	NA
Higher	76	12 (15.8)	1.75 (0.85–3.60)	NA
**Woman retained in care**				
Attended < 5 visits	708	64 (9.0)	1.00	1.00
Attended ≥ 5 visits	292	71 (24.3)	3.44 (2.32–5.08)	3.27 (2.14–4.98)
**Family able to cover expenses of tests**				
No	74	11 (14.9)	1.00	NA
Yes	338	61 (18.0)	1.26 (0.62–2.53)	NA
**Attended first post-test specialist consultation with partner**				
No	503	67 (13.3)	1.00	NA
Yes	497	68 (13.7)	1.05 (0.73–1.51)	NA
**Disclosed HBV-positive status to partner**				
No	114	6 (5.3)	1.00	1.00
Yes	886	129 (14.6)	3.10 (1.33–7.19)	2.81 (1.16–6.80)

CI: confidence interval; HBV: hepatitis B virus; NA: not applicable; OR: odds ratio.

Note: We included the following variables in the multivariable analysis: woman’s occupation, partner’s level of education, marital status, baby’s gestational age, partner’s age, woman’s age, attended first post-test specialist consultation with partner, family able to cover expenses of tests. In some instances the values do not add up to the sample size due to missing data.

### HBsAg status of family members

Among the 651 family members screened, 79 individuals (12.1%) tested positive for HBsAg, including 55 of 436 partners (12.6%; median age: 33 years; interquartile range, IQR: 29–38) and 24 of 215 children (11.2%; median age: 7 years; IQR: 4−12; Table 2). Of the 27 HBsAg-positive children, 15 children had a father screened for HBsAg, and three of these fathers (20.0%) also tested positive for HBsAg. In 13 HBsAg-positive children whose siblings were also tested, six children (46.0%) had another sibling positive for HBsAg, and in one household the father and the two children were carriers of HBsAg. 

In multivariable analyses, having a mother who was positive for HBeAg or who had HBV DNA level ≥ 200 000 IU/mL was significantly associated with a child being HBsAg positive (adjusted OR: 8.57; 95% CI: 2.49–29.48 and adjusted OR: 6.83; 95% CI: 1.61–29.00, respectively; Table 5). A larger family size was also associated with a higher risk of childhood HBV infection; children with at least four siblings had a 5.4 times higher risk of HBV infection (adjusted 95% CI: 1.40–20.77) than those with one to two siblings. Children aged 8 years or older had a higher prevalence of positive HBsAg (16/70 children; 22.9%) than those younger than 8 years (8/100 children; 8.0%), although this was not significant in the multivariable analysis. 

**Table 5:** Factors associated with hepatitis surface antigen positivity in the children of pregnant women with hepatitis B virus infection in Baskuy district, Ouagadougou, Burkina Faso, 2014–2019

Variable	Total no. of index children screened	No. (%) of children HBsAg positive	Crude OR (95% CI)	Adjusted OR (95% CI)
**Woman’s age at index child’s birth**				
One unit (year) increase in age	NA	NA	0.86 (0.77–0.95)	NA
**Index child’s age, years^a^**				
< 8	100	8 (8.0)	1.00	NA
≥ 8	70	16 (22.9)	4.40 (1.47–13.15)	NA
**No. of siblings of index child^b^**				
1–2	95	17 (17.9)	1.00	1.00
3	23	2 (8.7)	0.41 (0.12–1.49)	0.72 (0.17–3.06)
≥ 4	8	5 (62.5)	2.95 (1.00–8.70)	5.40 (1.40–20.77)
**Birth order of index child**				
1	7	2 (28.6)	1.00	NA
2	44	5 (11.4)	0.20 (0.04–0.97)	NA
3	35	4 (11.4)	0.16 (0.03–0.88)	NA
≥ 4	43	10 (23.3)	0.26 (0.05–1.40)	NA
**Woman’s HBeAg status **				
Negative	111	13 (11.7)	1.00	1.00
Positive	16	11 (68.8)	11.47 (4.42–29.82)	8.57 (2.49–29.48)
**Woman’s HBV DNA level, IU/mL**				
< 200 000	97	14 (14.4)	1.00	1.00
≥ 200 000	7	6 (85.7)	14.04 (4.90–40.28)	6.83 (1.61–29.00)
**Partner’s HBsAg status**				
Negative	87	14 (16.0)	NA	NA
Positive	14	5 (35.7)	1.37 (0.70–2.71)	NA

CI: confidence interval; HBeAg: hepatitis B e-antigen; HBsAg: hepatitis B surface antigen; HBV DNA: hepatitis B virus deoxyribonucleic acid assay: IU: international unit; NA: not applicable; OR: odds ratio.

^a^ Children 8 years or older were born in 1994–2006, before HBV vaccination was included in the expanded programme of immunization in Burkina Faso (year 2006).

^b^ Excluding the index child.

Notes: We included the following variables in the multivariable analysis: woman’s HBeAg status, woman’s HBV DNA level, number of siblings, child’s age, woman’s age at index child’s birth.

There was no association between women’s HBeAg status and partners’ HBV status. We also did not observe any association between the HBV status of the father and HBV infection in children (Table 6).

**Table 6:** Hepatitis B virus infection status of partners of pregnant women according to woman’s hepatitis B surface antigen carrier status and hepatitis B virus status of children, Burkina Faso, 2014–2019

Variable	Partner’s HBsAg status, no. (%) of men		*P* ^a^
	Negative	Positive	
**Woman’s HBeAg status**			
Negative	325 (88.6)	42 (11.4)	0.62
Positive	37 (86.0)	6 (14.0)	
**Woman’s HBV DNA level **			
< 200 000 IU/mL	300 (88.0)	41 (12.0)	1.00
≥ 200 000 IU/mL	34 (89.5)	4 (10.5)	
**Child’s HBsAg status**			
Negative	128 (84.8)	23 (15.2)	0.75
Positive	17 (81.0)	4 (19.0)	

HBeAg: hepatitis B e-antigen; HBsAg: hepatitis B surface antigen; HBV: hepatitis B virus; HBV DNA: hepatitis B virus deoxyribonucleic acid assay.

^a^ Fisher exact test.

## Discussion

Nearly half of the partners in this study agreed to have HBV screening, and the disclosure of women’s HBV status to her partner was important for successful screening; the uptake of screening was 46.4% and 21.9% in couples with and without disclosure, respectively. This finding agrees with previous HIV studies.^18^ The high rate of disclosure to the partners in our study might be due to the selective study population, since our analysis only included women who consented to be enrolled in the study cohort.^16^

In contrast to the partners, only a small proportion of children born to HBsAg-positive women were screened. This outcome might be because HBsAg-positive children tend to be asymptomatic and do not require any treatment. The clinical benefit of monitoring HBV-infected children, even in the absence of antiviral therapy, should be explained to their parents. In the multivariable analysis we found that maternal retention in care, maternal higher education level and the sharing of HBV status between the parents were significantly associated with higher screening uptake in children. These findings suggest the importance of better communication between health-care workers and parents to facilitate better understanding of hepatitis B disease. Another important aspect is the lack of subsidies to undertake HBsAg screening tests. Many people cannot afford the cost of testing in sub-Saharan Africa.^19^ Allocation of the financial resources for facilitating HBV testing in household contacts of HBV-infected women, particularly in children, should be considered.

In highly endemic settings, HBV transmission mostly occurred at birth or during childhood, especially before the widespread implementation of infant hepatitis B vaccination.^20^ In Burkina Faso, the prevalence of HBV core antibodies, a marker of previous exposure to HBV, has been reported to be 89.1% (214/240 individuals) in adults aged 18–60 years living in a rural area.^11^ Whether the partners of people with chronic HBV infection have higher prevalence of HBV than the general population remains to be debated. It is also controversial whether screening adults for HBsAg is an effective way to identify susceptible adults who would benefit from catch-up hepatitis B vaccination.^21^ WHO recognizes that in settings where the prevalence of HBsAg in the general population exceeds 2%, focused testing of high-risk populations alone will be insufficient to identify HBV-infected people. Testing of the general population is recommended instead.^2^ In our study, we found a high prevalence of HBsAg in partners of infected women (12.6%). In the 2010–2011 demographic and health survey in Burkina Faso, the HBsAg prevalence in men was 10.5% (723/6830 men; 95% CI: 9.6–11.4).^21^^,^^22^ Furthermore, in a study of 10 576 couples, a higher HBV seroprevalence was observed among individuals whose partners were infected (11.7%; 95% CI: 8.4–15.1) compared with those whose partners were not infected (8.1%; 95% CI: 7.4–8.9),^21^ suggesting that focused testing might be a more efficient way to find new cases than general population testing.

We also observed a relatively high HBsAg prevalence in children. This high prevalence can be explained by the fact that all these children have HBsAg-positive mothers and that birth dose hepatitis B vaccination was not provided as part of the expanded programme of immunization in Burkina Faso. The risk of mother-to-child transmission from HBV-infected mothers in the absence of any vaccination is high: about 40% from HBsAg-positive HBeAg-positive mothers and 5% from HBsAg-positive HBeAg-negative mothers in sub-Saharan Africa.^23^^,^^24^ Interestingly, we found a lower risk of HBsAg positivity in children younger than 8 years old who were born after 2006, the year when infant hepatitis B vaccination at 8, 12 and 16 weeks was introduced into the expanded programme of immunization in Burkina Faso. The rate of HBV infection in infants in African countries where HBV prophylaxis is based on vaccination starting at 6–8 weeks without neonatal immunoprophylaxis remains to be explored.^24^^,^^25^ A 2018 study, carried out in western Burkina Faso, showed that the risk of HBV infection in children remains substantial (9/265 children; 3.4%), despite a moderate vaccination coverage of 82.6% (219/265 children).^12^ The majority of these infected children had HBsAg-positive mothers, indicating the persistence of HBV mother-to-child transmission. Moreover, recent economic modelling has shown that adding monovalent HBV vaccine at birth would be cost-effective in Burkina Faso.^26^ Gavi, the Vaccine Alliance, recently published a strategic plan to support the implementation of birth dose vaccination in the first 24 hours of life using a monovalent vaccine in low-resource countries.^27^ While some West African countries, such as Senegal in 2016 and Benin in 2020, successfully integrated this birth dose vaccination into the expanded programme of immunization, in Burkina Faso the birth dose is scheduled for the year 2022.

In Côte d’Ivoire researchers found that in 154 infants without a birth dose who only received hepatitis B vaccination starting at 6 weeks of life, the risk of HBV mother-to-child transmission was negligible (0/132 infants) if their mothers were positive for HBsAg but negative for HBeAg.^28^ However, that study confirmed a substantial risk from HBeAg-positive mothers with a transmission rate of 59%. In our study, we observed a sixfold higher rate of HBsAg carriage in children born to HBeAg-positive women than in children born to HBeAg-negative women. It is well established that HBeAg-positivity and HBV DNA levels over 200 000 IU/mL during pregnancy are the main risk factors for HBV mother-to-child transmission.^16^ Furthermore, we found that in six out of 13 HBV-infected children another sibling also tested positive, suggesting that HBV infection in children might be clustered in the family of HBeAg-positive women. Having a large number of siblings was associated with a higher risk of HBV infection in children. This finding could be explained by a higher risk of horizontal transmission between children in larger households, as has been reported in Gambia and Senegal.^29^^,^^30^ There is also a greater chance of having an elder sibling born before the introduction of hepatitis B vaccination into the expanded programme of immunization, who is less likely to have been protected by immunization. Finally, there could be a higher frequency of maternal HBeAg carriage at the first childbirth due to the younger age of the women.^29^^,^^31^

To identify a large number of HBV-infected people who are not aware of their infection in sub-Saharan Africa, implementing mass HBV screening that targets the general population is appealing, but would pose considerable logistic and financial challenges. Moreover, a limited awareness of hepatitis B infection in the population may represent a barrier to the acceptance of an HBV diagnosis, linkage to care and lifelong treatment.^32^ Within the health-care resources of low-income countries, the antenatal consultation, generally accepted by the public, provides a unique and realistic opportunity to identify and link infected household contacts to hepatitis care.^32^ Although additional efforts should be made to increase screening uptake among partners and children, our study showed the feasibility of such a strategy in Burkina Faso.

Our study has limitations. Study participants were not representative of the whole of Burkina Faso, limiting the generalizability of the study findings to other contexts. The cost of the HBV testing was borne by households and not by the project. While this limitation could be a strength in estimating the uptake of HBV screening in a real-life setting, it is also a drawback to obtaining an unbiased estimate of HBV prevalence in the target population.

In conclusion, HBV testing in family members of women identified as carriers of HBsAg at antenatal care may be a promising approach for HBV diagnosis and linkage to care of exposed children and partners. Our study confirmed how sharing HBV status within couples is important for successful testing of partners and children for HBV. Children born before the introduction of hepatitis B vaccination, and those born to mothers with high viral load or viral replication markers were at a greater risk of HBV infection; these children should be prioritized for HBV screening and linkage to care.
